# Major Ampullate Spider Silk with Indistinguishable Spidroin Dope Conformations Leads to Different Fiber Molecular Structures

**DOI:** 10.3390/ijms17081353

**Published:** 2016-08-18

**Authors:** Justine Dionne, Thierry Lefèvre, Michèle Auger

**Affiliations:** Regroupement québécois de Recherche sur la Fonction, l'Ingénierie et les Applications des Protéines (PROTEO), Centre de Recherche sur les Matériaux Avancés (CERMA), Centre Québécois sur les Matériaux Fonctionnels (CQMF), Département de Chimie, Université Laval, Pavillon Alexandre-Vachon, Ville de Québec, QC G1V 0A6, Canada; justine.dionne.1@ulaval.ca (J.D.); michele.auger@chm.ulaval.ca (M.A.)

**Keywords:** Raman spectromicroscopy, spider silk fibers, spinning dope, silk protein structure, molecular orientation, orientation distribution function

## Abstract

To plentifully benefit from its properties (mechanical, optical, biological) and its potential to manufacture green materials, the structure of spider silk has to be known accurately. To this aim, the major ampullate (MA) silk of *Araneus diadematus* (*AD*) and *Nephila clavipes* (*NC*) has been compared quantitatively in the liquid and fiber states using Raman spectromicroscopy. The data show that the spidroin conformations of the two dopes are indistinguishable despite their specific amino acid composition. This result suggests that GlyGlyX and GlyProGlyXX amino acid motifs (*X* = Leu, Glu, Tyr, Ser, etc.) are conformationally equivalent due to the chain flexibility in the aqueous environment. Species-related sequence specificity is expressed more extensively in the fiber: the β-sheet content is lower and width of the orientation distribution of the carbonyl groups is broader for *AD* (29% and 58°, respectively) as compared to *NC* (37% and 51°, respectively). β-Sheet content values are close to the proportion of polyalanine segments, suggesting that β-sheet formation is mainly dictated by the spidroin sequence. The extent of molecular alignment seems to be related to the presence of proline (Pro) that may decrease conformational flexibility and inhibit chain extension and alignment upon drawing. It appears that besides the presence of Pro, secondary structure and molecular orientation contribute to the different mechanical properties of MA threads.

## 1. Introduction

Spider silk proteins (spidroins) represent the basic constituent of a range of natural filaments that are used to fulfill specific biological functions. Relying on its biocompatibility and remarkable mechanical properties, this raw material may become a promising resource for materials science. It may lead in the future to diverse and useful applications in various fields including biomedicine, optoelectronics, high-tech threads or textiles, particularly in a context where society’s sustainability requires, among other societal progresses, the development of green and durable technologies [1,2,3,4,5]. It is then desirable to reproduce natural silk in the laboratory and at industrial scale, or to produce silk-inspired materials with tailored characteristics. To achieve this goal, a deep understanding of the structure and formation of silk is necessary.

Silk threads are produced by spiders in specialized glands located in their abdomen. The archetype of silk is the dragline which is produced by the major ampullate (MA) gland and is thus termed MA silk. Its basic structural organization can be described by stiff crystalline nanodomains constituted by highly oriented polypeptide chain segments folded into β-sheets and distributed in an amorphous soft matrix composed of protein chains that adopt less oriented and more disordered secondary structures [6,7,8]. This semicrystalline architecture governs silk mechanical properties as it is influenced by the level of molecular orientation, the fraction of the crystalline phase, the limited size of the β-sheet nanocrystals, and by the hydrogen bonded network of the amorphous chains [9,10,11,12,13,14], among other factors.

The final material results from the molecular transformations of spidroins and their assembly during the course of the spinning process which is in turn generated by the mechanical constraints imposed by the spider. The sequence of the spidroin amino acid building blocks is segmented into successive motifs that undergo conformational and orientational changes during silk formation. MA spidroins include polyalanine (poly-Ala) segments which form the β-sheets [15,16] and glycine-rich segments GlyGlyX and GlyProGlyXX (GGX and GPGXX) (with *X* = Leu, Glu, Tyr, Ser, etc.) which constitute the disordered phase (3_1_-helices, turns) [17,18,19,20]. The MA fiber of orb-weaving spiders is actually made of two analogous spidroins which vary in proportion and slightly differ in sequence depending on the species, particularly in terms of proline (Pro) content. For example, in the case of *Nephila clavipes* (*NC*), the main protein (MaSp1, 80% mole) is Pro-free while MaSp2 is marked by the presence of Pro under the form of GPGXX motifs [21,22]. In the case of *Araneus diadematus* (*AD*), the two proteins (termed ADF3 and ADF4) [23] are both rich in Pro, leading to a higher global Pro content (16%, while *NC* contains only 3.5%) [24,25].

Major advances have been made to understand how spidroin primary structure (repetitive segment [26,27,28,29], non-repetitive N- and C-terminal regions [30,31]), spinning process (rheology [32,33], anatomy and ultrastructure of the gland [34,35] as well as physicochemical conditions of the lumen [36,37,38]) contribute to the formation of the final structure and to its properties. Among other factors, the role of Pro on the mechanical behavior of MA silk has been emphasized, especially since the discovery that the Pro content is positively correlated with the extent of supercontraction [13,39], a phenomenon by which silk fibers shrink longitudinally and swell radially upon exposure to water. The influence of Pro residues has been attributed to an increase in water plasticization due to a disordering effect on the polypeptide chains in the amorphous phase [13,39,40,41,42]. The improvement in the mechanics of MA silk upon evolution (extensibility, toughness) has been correlated with the appearance of the Pro-rich MaSp2 spidroins in the Orbiculariae clade [43].

**Figure 1:**
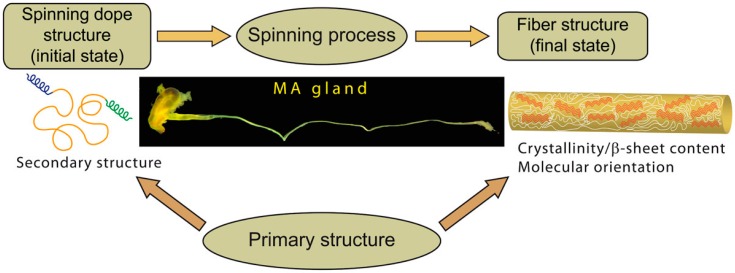
Schematics showing the relationships between the structure of major ampullate (MA) spidroins in the silk dope, the fiber structure, the spinning process and the primary structure. Silk formation corresponds to the transformation converting the spidroins in the dope (initial conformational state) into the silk fiber (final structural state). The spinning process (mechanical constraints applied, physicochemical environment) and primary structure (chemical composition) are the two factors that govern structural changes. On one hand, the spinning process triggers and induces conformational and orientational changes to the polypeptide chains. On the other hand, the response of spidroins and the final secondary structure they adopt are dictated by the sequence.

Silk thread structure is determined by the protein sequence characteristics and spinning process (Figure 1). The importance of both factors has been emphasized [26,39,44,45,46,47,48,49]. Whereas it may be inferred that silk structure is mainly “encoded” in the sequence (such that the spinning process may appear relatively unimportant), the MA gland environment and the way molecular transformations are induced are essential to lead to the appropriate fiber structure. Both factors are actually interconnected: the spinning process converts the spidroins conformation occurring in the sac of the MA gland into the highly organized structure of the fiber. However, the initial and final states as well as the response of the polypeptide chain to mechanical and physicochemical stimuli depend intimately on the sequence. The initial conformation may also by itself influence the molecular transformations that lead to the final structure. Overall, the relative contributions of the composition and architecture of the spidroins sequence on one hand and the spinning process on the other hand remains unclear.

To better understand the initial-to-final structure transition and the role of the sequence and spinning process, structural differences that may exist between MA silks from different spiders have to be evaluated in both the liquid (dope) and solid (fiber) states. The comparison of silks reeled in the same conditions and at the same speed may minimize the effect of the spinning process, thus potentially evidencing small structural differences due to the specificities of the spidroin’s primary structure. This is particularly relevant since the sequence, in particular the Pro content, can influence the conformation of the spidroins both in the dope and in the fiber.

Although tensile properties of MA silk of numerous spider species have been extensively investigated [13,39,50,51,52,53,54,55,56,57], researches devoted to comparisons of their structures are scarce. Even for the two most widely investigated spiders (*AD* and *NC*), data are lacking, in particular regarding the level of molecular orientation. A lower birefringence of *AD* with respect to *NC* has however been measured [13] and a higher level of random coil conformation has been proposed for *AD* from Raman spectromicroscopy [58]. A major advance has been made recently by a broad phylogenetic X-ray diffraction investigation of MA silk fibers [51]. Systematic data regarding the comparison of spidroin conformations in the MA dope of different species are inexistent.

The aim of this study is thus to compare quantitatively the molecular structure of *NC* and *AD* MA silk dopes and fibers. Raman spectromicroscopy has been used to investigate single monofilament and gland content and to determine the orientation level and secondary structure content. The present data suggest that besides the role of Pro residues, other molecular structural characteristics such as β-sheet content and molecular orientation have to be considered to exhaustively describe species-related differences in the mechanical properties of the MA spider silks. The sequence specificities also appear essential for the formation of the fiber structure.

## 2. Results

### 2.1. Spidroins Conformation in the Major Ampullate (MA) Dope

**Figure 2:**
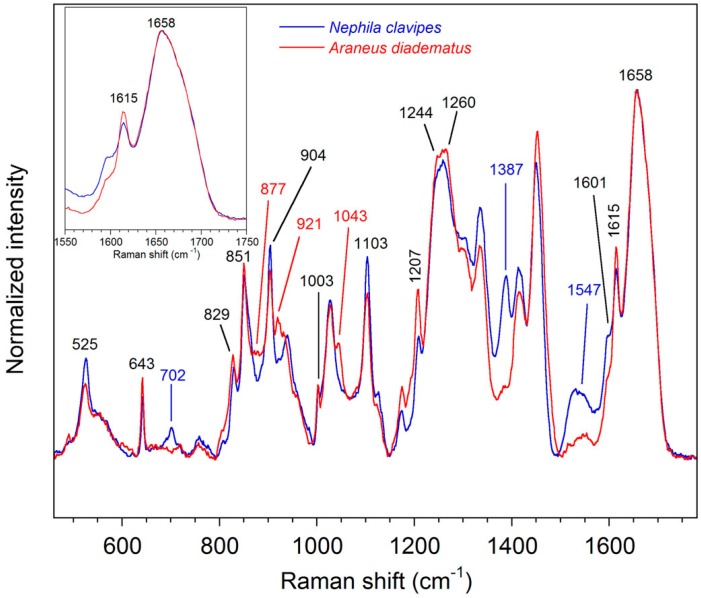
Raman spectra of the spinning dope contained in the sac of the *Nephila clavipes* (*NC*) and *Araneus diadematus* (*AD*) MA glands.

Raman spectra of the native silk dope of the *NC* and *AD* MA glands are shown in Figure 2. The normalized Raman intensity displays unique information about the chemical constituents of the spinning dopes. Since the silk dope is isotropic, Raman intensity is only influenced by the chemical composition and conformation of the samples. In other words, molecular orientation does not affect the Raman spectra. Some bands are conformation-sensitive whereas others are representative of the amino acid residues.

Bands representative of the secondary structure are mainly due to the peptide bonds, especially regarding the amide modes. The shape and position of the amide I bands (1658 cm^−1^) are superimposable for the two spider species as revealed by the inset scheme in Figure 2. This result indicates that the conformation of the spidroins is identical for *NC* and *AD*. As discussed elsewhere [16,59], these amide I bands reveal that the silk dope is essentially disordered with a small contribution from α-helix [60]. The amide III band is also very similar for both species, with two components at 1244 and 1260 cm^−1^ which are characteristic of disordered structure. The skeletal C_α_–C_β_ stretching band appears at 1103 cm^−1^ and supports the previous random coil assignment. The spectra also exhibit a peak at 525 cm^−1^ due to poly-Ala with an α-helical conformation. The lower intensity for *AD* indicates lower percentage of poly-Ala in the sequence.

Bands due to the amino acid side-chains allow discrimination between different silks. In particular, Pro bands at 877, 921, and 1043 cm^−1^ only appear in the spectrum of the *AD* dope. This was expected since, as previously mentioned, the MA spidroins of *AD* contain more Pro compared to *NC* [24,25]. Peaks due to tyrosine (Tyr) at 643, 829, 851, and 1615 cm^−1^ and to poly-Ala at 904 cm^−1^ are found in both spectra of liquid MA silk although that those related to Tyr are more intense for *AD*. Bands due to phenylalanine (Phe) residues arise at 1003 and 1601 cm^−1^ and the peak at 1207 cm^−1^ is characteristic of Phe and Tyr. A non-protein constituent previously identified as an isoquinoline compound [61] arises at 702, 1387, and 1547 cm^−1^ in the spectrum of *NC* [62].

### 2.2. Molecular Structure of the MA Fibers

#### 2.2.1. Qualitative Analysis

**Figure 3:**
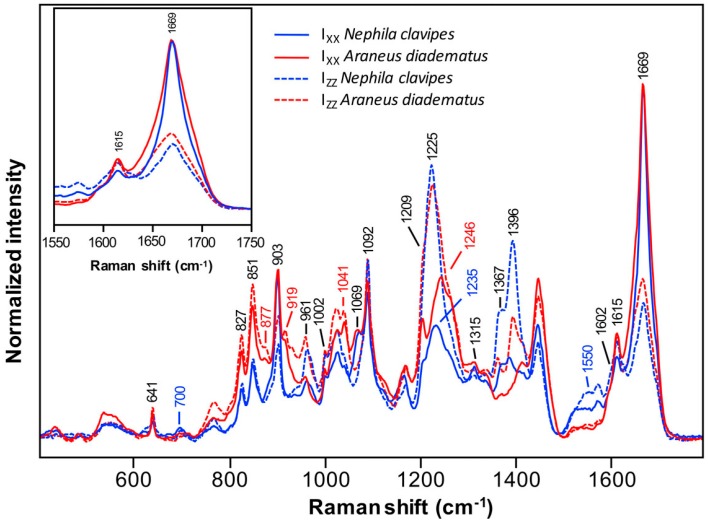
Polarized I_XX_ and I_ZZ_ Raman spectra of *Nephila clavipes* and *Araneus diadematus* MA silk fibers. Spectra are normalized so that the I_XX_ peak maximum is equal in the amide I region. They represent the mean spectra calculated over 12–14 series of measurements.

Figure 3 shows the polarized I_XX_ and I_ZZ_ spectra of the *NC* and *AD* MA threads. The complete series of polarized spectra are given in Figure S1. Unlike the dopes, silk fibers display anisotropy. Raman intensity thus depends not only on the conformation but also on molecular orientation. Regarding amino acid side-chains, the peaks due to Tyr at 641, 827, 851, and 1615 cm^−1^, to Phe at 1002 and 1602 cm^−1^, to Phe/Tyr at 1209 cm^−1^ and to poly-Ala at 903 cm^−1^ are still prominent for *AD* and *NC*. Consistently with the spectra of the liquid silk, the Pro bands at 877, 919, and 1041 cm^−1^ are only observed for *AD*. Peaks associated with isoquinoline in *NC* come out at 700 and 1550 cm^−1^. The band previously found at 1387 cm^−1^ overlaps with another vibrational mode due to the chain backbone at around 1396 cm^−1^ [63].

As confirmed by the positions of the I_XX_ amide I band at 1669 cm^−1^ and the amide III bands at 1225 and 1246 cm^−1^, both fibers exhibit a predominance of β-sheets. Other conformation-sensitive bands at 961, 1315, 1367, and 1396 cm^−1^ and skeletal C_α_–C_β_ stretching at 1069 and 1092 cm^−1^ confirm that the β-sheet conformation is preponderant for the two spiders. The bands at 1367 and 1396 cm^−1^ are more intense in the I_ZZ_ spectrum of *NC* compared to *AD*, which suggests that *NC* MA silk contains more β-sheets than *AD*. However, a component of the isoquinoline contaminant described earlier contributes to the band at 1396 cm^−1^. Furthermore, the I_XX_ amide III band of *NC* is less intense and is located at 1235 cm^−1^ rather than at 1246 cm^−1^ for *AD*. This difference is explained below.

The variations of band intensities with polarization indicate that the proteins in MA filaments exhibit a preferential orientation. The β-sheets of MA fiber are known to be oriented along the fiber axis [64,65]. Consequently, the carbonyl groups of the peptide bonds, which are principally responsible for the amide I signal, are mainly perpendicular to the fiber axis (Figure S2). A higher intensity of the amide I band in the I_XX_ spectrum is then expected over the I_ZZ_. For comparison purposes, the I_XX_ amide I bands of all series of spectra have been normalized to unity. Consequently, the weaker the I_ZZ_ amide I band, the higher the orientation of the fiber. Conversely, a stronger I_ZZ_ amide III band, or a weaker I_XX_ amide III band, means a higher orientation of the chains since the amide III band is mostly due to the C–N stretch of the peptide bond which is oriented along the fiber axis in β-sheets [66].

Figure 3 shows that the I_ZZ_ amide I band of *NC* is weaker and its I_ZZ_ amide III band is more intense as compared to *AD* (its I_XX_ amide III band is also weaker). Those observations suggest that the *NC* MA spidroins are more oriented than those of *AD*. Furthermore, the inset illustration in Figure 3 reveals that the amide I band of *AD* is broader compared to *NC*. This observation indicates that the β-sheets of the *AD* fiber are less oriented and/or less abundant than for *NC*.

#### 2.2.2. Secondary Structure Content

**Figure 4:**
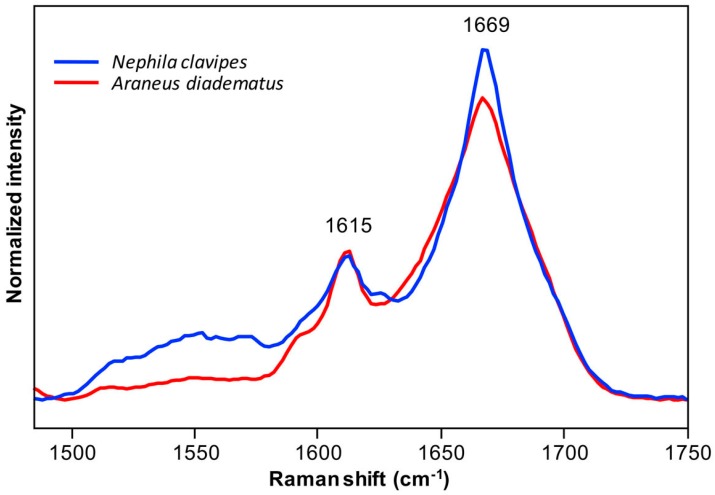
Orientation-insensitive spectra in the amide I region calculated from the average polarized Raman spectra of *Nephila clavipes* and *Araneus diadematus* fibers shown in Figure 3 and Figure S1. The spectra are normalized with respect to the total area of the amide I band.

Figure 4 presents the orientation-insensitive spectra of *NC* and *AD* MA fibers in the amide I region calculated from the average polarized spectra. The Raman intensity of these spectra is only representative of the abundance of spidroin conformational elements, so that they can be used to evaluate the amount of secondary structures. Since the peak maximum of the amide I band at 1669 cm^−1^ is higher for *NC* than for *AD*, it appears that the *NC* MA silk contains more β-sheets. Alternatively, the spectrum of *AD* is broader compared to *NC*, especially at around 1645 cm^−1^, an observation that suggests that disordered structures are more abundant for the *AD* silk.

**Figure 5:**
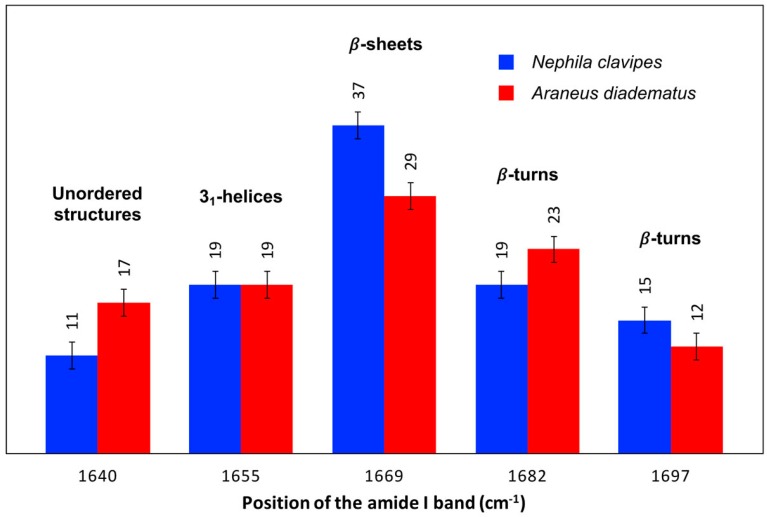
Relative band areas of the different amide I components of *Nephila clavipes* and *Araneus diadematus* fibers. The approximate error on the secondary structure content due to curve-fitting is ±3% and is represented by the error bars on the figure.

The percentage of each secondary structure that contributes to the amide I band has been further estimated by curve-fitting (Figure 5). Consistently with the above qualitative analysis, the β-sheets are the predominant conformation in both MA fibers, the β-sheets being more prevalent in *NC* (37%) than in *AD* (29%) silk. The *AD* filament contains more disordered structures which is in agreement with previous data [58]. A slight difference in the amount of β-turns at 1682 cm^−1^ is also found between the *AD* (23%) and *NC* (19%) threads. The approximate error on the band areas being 3%, this distinction between the two species appears minor. Finally, *NC* and *AD* MA fibers have the same relative abundance of 3_1_-helices (19%) and β-turns at 1697 cm^−1^ (12%–15%).

#### 2.2.3. Quantitative Orientation Analysis

The molecular orientation of the *NC* and *AD* fibers was characterized using the polarized amide I vibration bands (mainly due to the C=O stretching vibration). As this band is dominated by the β-sheet component, it is assumed that the orientation mainly reflects peptide bonds adopting a β-sheet conformation in the threads. The peak maximum of the amide I band was used to calculate the qualitative orientation parameter *R*′ and the order parameters *P*_2_ and *P*_4_ from the intensity ratios *R*_1_ and *R*_2_. MA silk samples have a weaker I_ZZ_ intensity in the amide I region, i.e., *R*′ < 1 and *P*_2_ < 0. A more negative value of *R*′ = 1 − (*I*_XX_/*I*_ZZ_) indicates a higher degree of orientation of the β-sheets. Similarly, the more negative the *P*_2_ value, the higher the molecular orientation.

**Figure 6:**
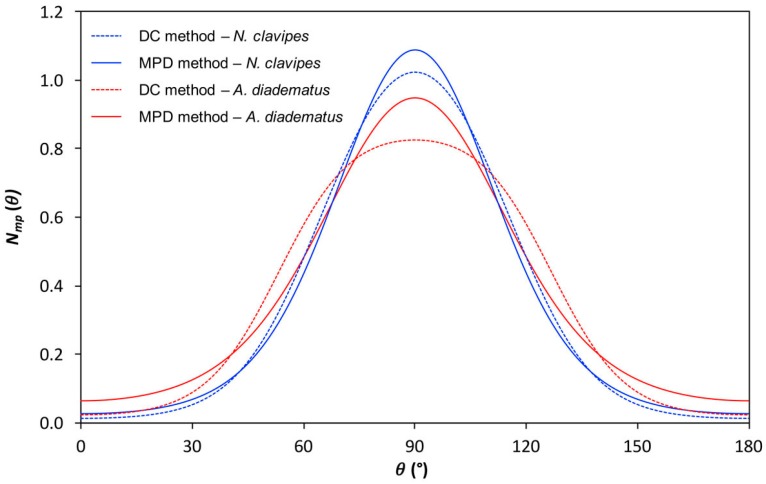
Most probable orientation distribution (MPD) functions, *N*_mp_(θ), of the carbonyl groups as determined from the order parameter values (Table 1) of *Nephila clavipes* and *Araneus diadematus* MA silk fibers according to the MPD and depolarization constant (DC) methods.

The parameter *R*′ as well as *P*_2_ and *P*_4_ for *AD* and *NC* threads are presented in Table 1. *R*′ values of −1.63 and −1.24 were found for *NC* and *AD* fibers, respectively, which confirm that the *NC* silk is more oriented than *AD*. This qualitative result is confirmed by the quantitative *P*_2_ and *P*_4_ values calculated with the DC (depolarization constant) method (see the Experimental section). The *P*_2_ values are negative due to the fact that the carbonyl groups are mainly perpendicular to the fiber axis. With the DC method, the *P*_2_ value of the MA silk of *NC* is lower (more negative) than for *AD* indicating that *NC* spidroins are more oriented than *AD* ones. The most probable distribution functions *N*_mp_(θ) estimated from the order parameters and the information theory is shown in Figure 6. The full width at half maximum (FWHM) is 57° for *NC* and 75° for *AD*. The distribution of orientation is thus narrower for *NC* than for *AD*, showing quantitatively that the level of orientation of MA silk is higher for *NC*. The maximum of the function appears at 90° which is consistent with a perpendicular orientation of the structural units (carbonyl groups) for both species.

As can be seen, the top of *N*_mp_(θ) of *AD* is flattened, which actually seems not physically realistic. This flaw may be due to uncertainties on the intensity ratios and/or accuracy of the depolarization ratio. The values of order parameter *P*_4_ is particularly sensitive to such variations and, as noticed previously [67], small differences in *P*_4_ values can lead to significant differences in the orientation distributions. A second method, called the MPD (most probable distribution) method (see the Experimental section), assumes that the orientation distribution is Gaussian and has thus been tested to determine *P*_2_ and *P*_4_. The two methods give closely the same *P*_2_ but different *P*_4_ values (Table 1). The shape of *N*_mp_(θ) is not strongly affected by the calculation method for *NC*, but more important changes are observed for *AD*. As the orientation distribution functions of the two species are Gaussian with the MPD method, they are more appropriately compared. The FWHM calculated with the MPD method is 51° for *NC* and 58° for *AD* confirming that *NC* spidroins are more oriented than those of *AD* silk.

### 2.3. Sequences Analysis

**Figure 7:**
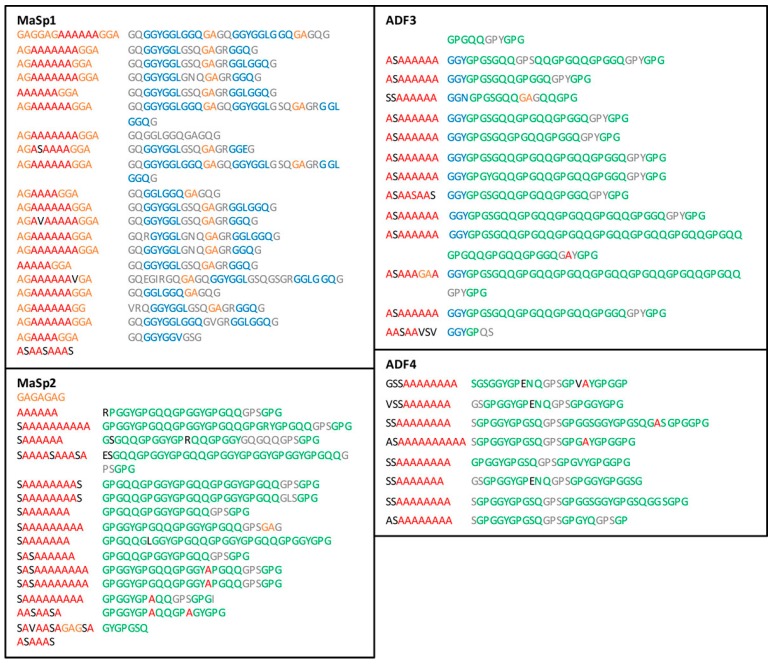
Amino acid sequences for the spidroins composing the MA gland silk of *Araneus diadematus* (ADF3 and ADF4) [23] and *Nephila clavipes* (MaSp1 and MaSp2) [21,22]. Single letter code for amino acids is used to simplify the analysis. The A_n_, GA, GGX, and GPGXX motifs are inspired from those of Gatesy et al. [44] and are indicated in red, orange, blue, and green respectively, where X represents a small subset of amino acids (*X* = L, Q, T, S, etc.). Amino acids that have undergone substitution in the motifs are colored in black and those that do not contribute to any motifs are colored in grey.

The amino acid sequences of the spidroins forming the MA silk are presented in Figure 7. ADF3, ADF4, and MaSp2 all belong to spidroin-2 type [23] and exhibit the same motifs: blocks of A_n_ and GA with a predominance of GPGXX motif. The spidroin-1 type MaSp1 is quite different with a prevalence of GGX motif, more A_n_-GA regions and an absence of Pro. *AD* and *NC* also exhibit similar length of poly-Ala. Since *AD* MA silk seems to be composed of ADF3 and ADF4 proteins in a 3:2 ratio [23], the Pro content estimated from the sequence is about 15% whereas *NC* MA silk contains 3% of Pro since MaSp1 accounts for 80% of the total protein content [13]. These Pro percentages are consistent with those determined by chemical methods [24,25].

The proportion of poly-Ala estimated from the sequence, including the GA motifs [18,20], is 34% for *NC* and 23% for *AD*. This difference is consistent with the lower intensity of the poly-Ala band at 525 cm^−1^ for *AD* silk dope. Assuming that the poly-Ala regions form the β-sheets [15,17,68], there is a good agreement between the β-sheet content evaluated from the Raman and sequence analyses. There is a small discrepancy between both methods (Raman analysis gives higher values than the sequence analysis), but the amount of β-sheets seems to be roughly predictable from the poly-Ala content.

## 3. Discussion

### 3.1. The Conformation of the Spidroins in the Dope Is Not Critical for the Fiber Structure

The amino acid composition of the *NC* and *AD* MA silks are similar but distinct, the former fiber being mainly composed of the Pro-deficient MaSp1, the latter being formed of two Pro-rich MaSp2-like spidroins. These different chemical compositions do not lead to distinctive conformation in the sac of the MA gland. This observation is consistent with the fact that recombinant MaSp1 and MaSp2 spidroins do not reveal conformational differences in solution by vibrational spectroscopy [69]. It may then be hypothesized that the sequence difference is not determinant for the initial spidroin conformation. Overall, vibrational bands of the backbone show that for both species the secondary structure is typical of disordered proteins, with a minor contribution of α-helix.

Contrarily to the dope, the spidroins of the two spiders exhibit clear molecular differences in the fibers, although they share the same structural pattern. It may thus be suggested that the initial conformation in the dope is relatively unimportant with regards to the final structure of MA threads. The molecular characteristics of the dope (disordered chains involved in 3_1_-helical structures without intramolecular bonds) may however promote or be important for the efficient folding of the polypeptide chains into oriented β-sheets in the fiber, but some details of the final structure (accurate β-sheet content and level of orientation) may be modulated by the sequence and the spinning process.

The fact that the spidroin conformations of *NC* and *AD* exhibit disparities in the final state but not in the initial state suggests that sequence specificities are more extensively expressed in the solid than in the solution state. The intrinsic secondary structure propensity of *NC* and *AD* MA spidroins thus appear identical in solution. In particular, the GGX and GPGXX motifs, that are predominant in *NC* and *AD* MA silk, respectively, seem to be conformationally equivalent in the dope. This may mostly be due to the chain flexibility in the aqueous environment (high conformational freedom), in particular to the prevalence of interactions between polypeptide chains and water (i.e., the absence of chain-chain interactions). After the spinning process, when the polypeptide chains are in close proximity and the intermolecular interactions maximized, the sequences seem to express their divergent secondary structures in the silk fiber.

### 3.2. Molecular Orientation and β-Sheet Content Influence Silk Mechanical Properties

The MA silks of the *Nephila* and *Araneus* genera differ in their tensile properties, the latter fibers being generally tougher and more extensible. Although mechanical differences have initially been observed in the dry state [52,55,56,70], they appeared more recently not to be so clear when comparing *NC* and *AD* [13]. By contrast, distinctions in the mechanical behavior seem to be particularly marked in the wet (maximum supercontracted) state [13].

In recent years, deviation in the mechanical properties of the MA silk with species has been accounted for by the Pro content, for example by the fact that the *AD* fiber is composed of two MaSp2-like proteins whereas that of *NC* is mainly made of MaSp1 [13,41,71]. The presence of Pro is expected to prevent the formation of regular secondary structure (mainly β-sheets), tight packing of the chains, and intermolecular H-bonding [13,39,41,42]. Contrarily, the Gly motifs, which are predominant in *NC*, would be more appropriate to form regular structures and an ordered organization [13].

The present data show that other structural parameters have to be taken into consideration when comparing MA silks. Molecular orientation and β-sheet content exhibit significant difference depending on the species. These two parameters obviously can strongly influence tensile properties. Their influence on MA silk properties is in line with findings regarding flagelliform silk. The large extensibility of this thread has long been related to the predominance of the GPGXX motif [72,73]. However, this silk also contains a small proportion of oriented β-sheets, which *de facto* includes this fiber into the archetypal MA silk family [74]. Thus, the increase in the breaking stress and breaking strain of this silk observed with different species seems to be related to higher β-sheet content and molecular alignment [74].

The amount of β-sheet of the *NC* and *AD* MA silk fibers can confidently be rationalized from the proportion of poly-Ala and GA motifs of the sequence, suggesting that the secondary structure content is in large part determined by the primary structure and is thus slightly influenced by the spinning process (although it is necessary to induce the conformational conversion towards β-sheets). The estimation of the β-sheet content by Raman spectromicroscopy and primary structure show that β-sheets are more abundant in *NC* than in *AD* MA silk. The lower β-sheet content of the *AD* MA silk, i.e., the higher proportion of amorphous phase, is likely to contribute to the higher extensibility of the *AD* MA silk with respect to *NC*. It has been proposed that the presence of Pro (under the form of GPGXX motifs) may globally promote disordered structures, which in turn may make the MA fiber more flexible [13,39,43,73]. However, the present results indicate that the β-sheet content, and reversibly the global amount of disordered chains, is not affected by Pro residues. Pro may rather make the *AD* amorphous phase more disordered than that of *NC*, which may in turn affect the tensile properties of these MA silks. Since the present results and conclusions have been obtained from only two spider species, they need to be generalized to a broader range of MA silks.

The Raman data also show that the level of molecular orientation of MA silk is higher for *NC* than for *AD*, which is in agreement with birefringence measurements [13]. This structural characteristic may also contribute to the fact that the fiber is more brittle for *NC* than for *AD*. Since the MA silks investigated here have been reeled at the same speed, it may be questioned whether the different levels of orientation are determined by the spinning process. More precisely, the difference may arise from the different physicochemical composition, differences in the gland anatomy or, more probably, rheological properties of the dope. However, no data such as spidroin concentration in the dope actually supports such an assumption. A more likely hypothesis lies in the fact that the sequence may be at the origin of these structural differences. As a matter of fact, due to its pyrrolidine ring structure, Pro residues may limit the conformational flexibility of the chain, thus inhibiting chain extension and molecular alignment upon drawing.

## 4. Materials and Methods

Adult *NC* and *AD* females were collected in Florida (USA) and Québec (Canada), respectively. They were raised in the laboratory at 24 ± 2 °C and 58% ± 5% relative humidity (RH) and fed with small crickets and 10% *w*/*v* glucose solution. For the analysis of liquid silk, the MA glands were extracted, deposited on glass slides or polystyrene petri dishes and immersed in a phosphate saline buffer. The epithelium was gently removed or pierced to expose the native liquid silk to the laser beam. Great care was taken to perturb minimally the silk material during the dissection procedure. In order to obtain fibers by forced reeling, spiders were anesthetized with CO_2_ and fixed on a support. The MA silk was then reeled on 1.3-cm diameter test tubes from awaken spiders at 1 cm/s. MA silks were stored hidden from sunlight to avoid degradation.

About 5-cm long monofilaments were gently fixed on glass slides with one-sided tape at its extremities and at different locations along the fiber. The spectra were recorded at 22.0 ± 0.5 °C and under 20% ± 5% RH using a LabRam 800HR Raman spectrometer (Jobin Yvon Horiba, Villeneuve d’Ascq, France) coupled to an Olympus BX 30 motorized stage microscope. The 514.5-nm line of an argon-ion laser (Coherent, INNOVA 70C Series Ion Laser, Santa Clara, CA, USA) was used as excitation light. The laser beam was focused by a 100× objective (*Numerical Aperture* (*NA*) = 0.9, Olympus, Richmond Hill, ON, Canada) generating an intensity of 3–5 mW at the sample. The confocal hole and the entrance slit of the monochromator were fixed at 400 μm and 200 μm, respectively. A 600 lines/mm holographic grating was used to disperse the different wavenumbers of the samples on the one-inch open electrode Peltier-cooled CCD detector (1024 × 256 pixels) (Andor Technologies, Belfast, Northern Ireland).

Spectra of the silk dope were recorded in the dry state as it has been shown that they are virtually identical to those obtained in the hydrated state [16]. Five spectra were recorded at different points on two distinctive dope samples. Orientation and secondary structure of the fibers were determined from the amide I band using linearly polarized light. A half-wave plate (Melles Griot, Carlsbad, CA, USA) was used to change the polarization of the incident light either perpendicular (x) or parallel (z) to the fiber axis (Figure S2). A polarizer was placed before the entrance slit of the monochromator to orient the polarization of the scattered light along the x or z direction. A broad-band quarter-wave plate was also used after the polarizer to eliminate the polarization dependence of the grating. Since the system works in a backscattering configuration, it allows the acquisitions of four independent polarized spectra (thereafter called a “series”) with an acquisition time of 2 × 30 s and identified as I_XX_, I_XZ_, I_ZZ_, I_ZX_ (the first index corresponds to the incident light, the second to the scattered light). To ensure that the focus was constant through the acquisitions, i.e., that non-desired intensity variation occur during measurements, a second I_XX_ spectrum was collected at the end of the series and compared with the first I_XX_ measurement. No sign of sample deterioration was observed under these experimental conditions.

Spectra treatments were all performed using GRAMS/AI 7.0 (ThermoGalactic, Salem, NH, USA). No smoothing was applied on the spectra. A cubic baseline was subtracted to correct the fluorescence background over the spectral range of 400–1800 cm^−1^. To take into account the polarization dependence of the instrument, correction factors were calculated from the totally depolarized band of liquid chloroform at 262 cm^−1^, which were then applied on the spectra of silk samples. For each series, the peak maximum of the amide I band I_XX_ spectra was normalized to unity and the other polarized spectra normalized accordingly. The spectra were aligned along the wavenumbers axis using the tyrosine band at 1615 cm^−1^. For the present study we investigated three fibers, probed 1–3 points on each fiber and measured 1–3 series of polarized spectra on each point. The overall standard deviation on the measurement of the amide I intensity ratios is lower than 0.03. Averaged polarized spectra were obtained from 12–14 series for each spider species over three different fibers.

For the determination of the secondary structure, orientation-insensitive spectra were calculated from a linear combination of the average polarized spectra [75]. The spectral decomposition of the amide I band was then carried out accordingly to the method previously described [59]. Briefly, a linear baseline was first subtracted in the amide I region (1490–1750 cm^−1^). The amide I band decomposition was carried out with five components located at 1640 (unordered structure), 1655 (3_1_-helix), 1669 (β-sheet), 1682 (turn), and 1697 (turn) cm^−1^, respectively [59]. Mixed Lorentzian and Gaussian functions were used. Curve-fitting calculations were implemented using the initial band parameters (positions, widths and shapes) optimized previously for other MA silks with constraints on the variations of the parameters [59]. Band area of each component was divided by the total area of the amide I band to determine the structure content.

Molecular orientation was estimated qualitatively from the parameter *R*′ defined as *R*′ = 1 − (*I*_XX_/*I*_ZZ_). Positive and negative values of *R*′ of the amide I band indicate that the polypeptide chains are mainly parallel and perpendicular to the fiber axis, respectively. *R*′ = 0 correspond to an isotropic sample. Molecular orientation was also assessed quantitatively in terms of order parameters *P*_2_ and *P*_4_ [76]. *P*_2_ follows the same interpretation rules than *R*′ but values are limited between −0.5 and 1. The method to determine the order parameters *P*_2_ and *P*_4_ from polarized Raman measurements was first described by Bower (1972) [77]. It was extended to Raman spectromicroscopy (i.e., in backscattering configuration with four spectra) by Turrell and coworkers [78,79], and adapted to the amide I tensor (assuming a cylindrical symmetry) by Rousseau et al. [80]. This latter method, referred to as depolarization constant (DC) method by Richard-Lacroix et al. [67], requires the determination of the shape of the Raman tensor from an isotropic sample with the same chemical composition as the oriented sample, assuming that the depolarization ratio is identical for both samples. For silk, this ratio has previously been determined to be 0.21 ± 0.01 [80]. The details of the determination of *P*_2_ and *P*_4_ are determined from two polarized intensity ratios, noted *R*_1_ = *I*_ZX_/*I*_ZZ_ and *R*_2_ = *I*_XZ_/*I*_XX_, and are given elsewhere [80]. The order parameter values and the associated standard deviations were calculated from the mean intensity ratio values of each (three) fiber.

A variant of the DC method, called the most probable distribution (MPD) method, was proposed by Richard-Lacroix et al. [67]. This procedure eliminates the determination of the depolarization ratio and instead assumes that the orientation distribution is Gaussian. This assumption in turn provides a relationship between *P*_4_ and *P*_2_, which then allows their determination using the two ratios *R*_1_ and *R*_2_. Finally, the distribution of orientation was estimated by both methods by calculating the most probable distribution of orientation (*N*_mp_(θ)) from the values of *P*_2_ and *P*_4_ using the information entropy theory [81,82,83,84].

## 5. Conclusions

This work provides a structural comparison between the MA dope and fiber silk of the two widely studied spiders, *NC* and *AD*. Quantitative values of the secondary structure contents and orientation of the MA spidroins have been determined. The *AD* MA fiber exhibits lower structural and orientational orders compared to the *NC* thread. Such structural differences certainly contribute to the mechanical properties of these MA silks. Secondary structure (i.e., β-sheet content) of the fiber seems to be mainly driven by the amino acid sequence (poly-Ala content). It is proposed that Pro residues (partly) inhibit molecular alignment of *AD* MA spidroins during the spinning process. The differences in the sequence of *NC* and *AD* spidroins do not result in distinctive conformation in the dope solution. This identical spidroin conformation suggests that it is not a decisive factor for the formation of specific fiber structure. Since differences in the mechanical properties of MA silk fibers are more obvious after supercontraction [13,85], it appears necessary to examine the orientation level and secondary structure of these two silks in this state. Moreover, details regarding the effect of reeling speed on silk molecular structure may also be informative to better understand the effect of the spinning process on the orientation level.

## Figures and Tables

**Table 1:** Qualitative parameter *R*′ = 1 − (*I*_XX_/*I*_ZZ_) and order parameters *P*_2_ and *P*_4_ of *Nephila clavipes* and *Araneus diadematus* major ampullate (MA) silk fibers as estimated from the polarized amide I bands and evaluated according to the depolarization constant (DC) and most probable distribution (MPD) methods.

Species	*R*′ *	*P*_2_ *		*P*_4_ *	
		DC Method	MPD Method	DC Method	MPD Method
*N. clavipes*	−1.63 ± 0.008	−0.306 ± 0.005	−0.307 ± 0.005	0.075 ± 0.005	0.089 ± 0.004
*A. diadematus*	−1.24 ± 0.008	−0.256 ± 0.007	−0.258 ± 0.006	0.02 ± 0.02	0.059 ± 0.003

* The order parameter values and the associated standard deviations were calculated from the mean intensity ratio values of each (three) fiber.

## Supplementary Materials

Supplementary materials can be found at www.mdpi.com/1422-0067/17/8/1353/s1.
