# Supplementary material for: Major Ampullate Spider Silk with Indistinguishable Spidroin Dope Conformations Leads to Different Fiber Molecular Structures
Source: Int J Mol Sci. 2016 Aug 18;17(8):1353. doi: 10.3390/ijms17081353 (PMC5000749; doi:10.3390/ijms17081353)
Supplement: Supplementary file 1 [file ijms-17-01353-s001.pdf]

Supplementary Materials: Major Ampullate Spider Silk with Indistinguishable Spidroin Dope Conformations Lead to Different Fiber Molecular Structures

Justine Dionne, Thierry Lefèvre and Michèle Auger

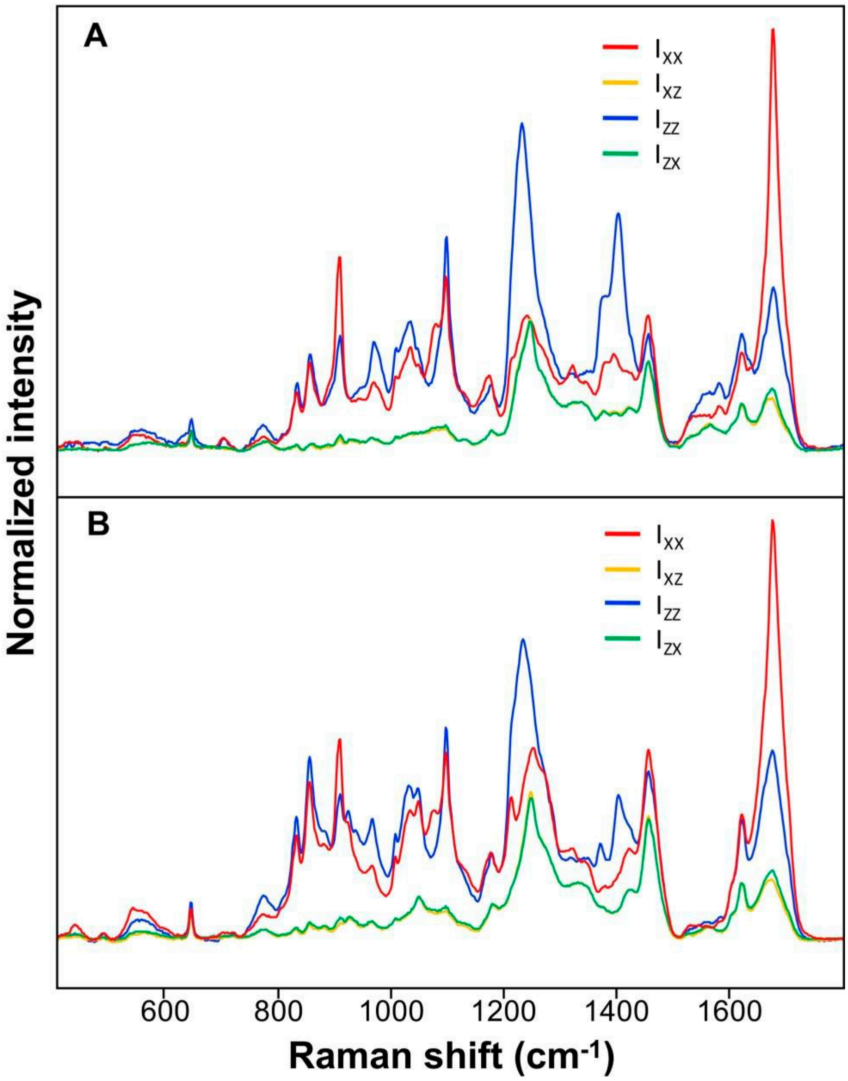

Figure S1. Polarized Raman spectra of (**A**) *Nephila clavipes* and (**B**) *Araneus diadematus* MA silk thread.

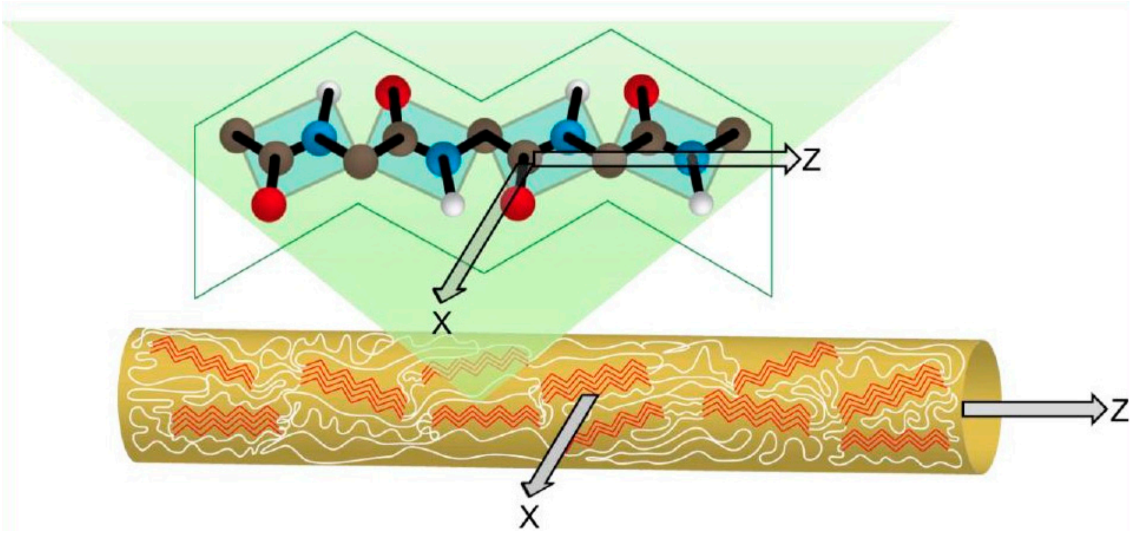

Figure S2. Representation of the coordinate system used, the alignment of the fiber for the Raman experiments (**down**) and the corresponding orientation of the β-sheets and carbonyl groups (**top**). Grey, blue, red and white spheres represent carbon, nitrogen, oxygen and hydrogen atoms of the polypeptide chains, respectively. Red structures in the fiber represent β-sheets while white lines symbolize the amorphous matrix of the silk fiber.
